# Predicted Public Health and Economic Impact of Respiratory Syncytial Virus Vaccination with Variable Duration of Protection for Adults ≥60 Years in Belgium

**DOI:** 10.3390/vaccines11050990

**Published:** 2023-05-16

**Authors:** Maarten J. Postma, Chih-Yuan Cheng, Nasuh C. Buyukkaramikli, Luis Hernandez Pastor, Ine Vandersmissen, Thierry Van Effelterre, Peter Openshaw, Steven Simoens

**Affiliations:** 1 Department of Health Sciences, Unit of Global Health, University Medical Center Groningen, University of Groningen, 9713 AV Groningen, The Netherlands; 2 Department of Economics, Econometrics & Finance, Faculty of Economics & Business, University of Groningen, 9749 AE Groningen, The Netherlands; 3 Janssen Pharmaceutica NV, 2340 Beerse, Belgium; 4 Janssen-Cilag NV, 2340 Beerse, Belgium; 5 National Heart and Lung Institute, Imperial College London, London SW3 6LY, UK; 6 Department of Pharmaceutical and Pharmacological Sciences, KU Leuven, 3000 Leuven, Belgium

**Keywords:** respiratory syncytial virus, vaccine, older adults, health economic evaluation

## Abstract

Respiratory syncytial virus (RSV) is a leading cause of acute respiratory infection (ARI) in older adults. This study used a static, cohort-based decision-tree model to estimate the public health and economic impact of vaccination against RSV in Belgians aged ≥60 years compared with no vaccination for different vaccine duration of protection profiles from a healthcare payer perspective. Three vaccine protection durations were compared (1, 3, and 5 years), and several sensitivity and scenario analyses were performed. Results showed that an RSV vaccine with a 3-year duration of protection would prevent 154,728 symptomatic RSV-ARI cases, 3688 hospitalizations, and 502 deaths over three years compared to no vaccination in older adults and would save EUR 35,982,857 in direct medical costs in Belgium. The number needed to vaccinate to prevent one RSV-ARI case was 11 for the 3-year duration profile, while it was 28 and 8 for the 1- and 5-year vaccine duration profiles, respectively. The model was generally robust in sensitivity analyses varying key input values. This study suggested that vaccination could substantially decrease the public health and economic burden of RSV in adults ≥60 years in Belgium, with benefits increasing with a longer duration of vaccine protection.

## 1. Introduction

Respiratory syncytial virus (RSV) is a leading cause of acute respiratory infection (ARI) [1]. Older adults and adults with comorbidities or immunosuppression experience higher rates of hospitalizations and emergency department visits and more frequent and severe complications than healthy and younger adults [2,3,4,5]. Additionally, older adults hospitalized with RSV infections may experience higher rates of morbidity and mortality compared to influenza [6].

RSV in Belgium demonstrates a seasonal transmission pattern with higher incidence rates between September and March of the following year [7]. Of two prospective multi-country studies that included Belgian cohorts of adults ≥60 years, one estimated the seasonal incidence of RSV illness in community-dwelling elderly at 4.2–7.2% [8]. The other indicated that the prevalence of RSV was significantly higher and the course of illness was worse in primary care patients aged ≥60 compared to younger adults (18–59 years) [9].

The economic burden of RSV disease in older adults is also high. In the US, annual direct healthcare costs due to RSV in adults ≥60 years total USD 3.9 billion [10]. No similar study is found thus far estimating total RSV-related direct medical costs in Europe.

Increased awareness of the significant RSV burden in older and high-risk adults [11] has led to the development of new vaccines [12,13,14,15]. It is therefore important to understand the public health and economic impact of vaccines against RSV in older adults.

The objective of this study was to evaluate the public health and economic impact of vaccination against RSV-related disease compared to no vaccination in older adults (≥60 years) from the healthcare payer perspective in Belgium. Studies in similar populations assessed the potential clinical and economic impact of RSV vaccination [16,17], assuming a vaccine protection duration of one RSV season, whereas this study examined the impact of vaccines with varying durations of protection against RSV disease and RSV-related hospitalizations.

## 2. Materials and Methods

### 2.1. Modeling Approach

A cohort-based model was developed in Microsoft Excel (Microsoft 365 Apps for Enterprise) to estimate the expected public health and economic outcomes of vaccination versus no vaccination in adults ≥60 years in Belgium from the Belgian healthcare payer perspective. The model follows up the simulated population over a time horizon reflecting the vaccine duration of protection, varying from one to five years (see Section 2.2). The model is static, meaning that the rate at which susceptible older adults become infected remains constant over time, without indirect herd protection from vaccination. While RSV transmission is expected across age groups [18], the current model focuses on the potential vaccine impact resulting from the vaccinated population of ≥60-year-olds. The model further assumes that an individual can acquire at most one RSV infection per year. A decision tree model was chosen given its suitability to model interventions against acute RSV disease with distinct outcomes that can be measured at a specific point in time [19].

**Figure 1:**
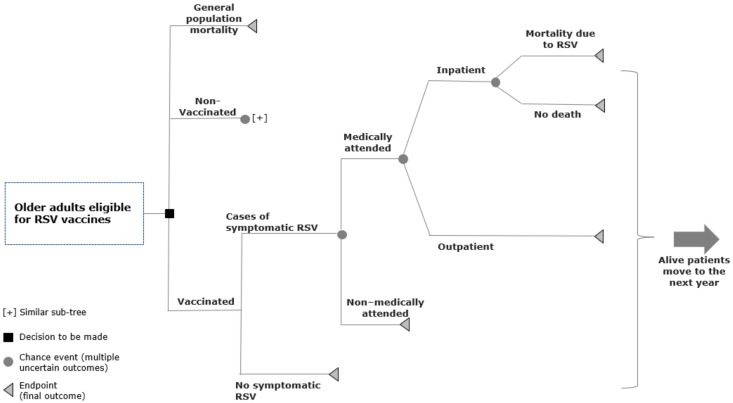
Model structure featuring no vaccination versus vaccination.

The model structure (Figure 1) features two arms: vaccination and no vaccination. Individuals in the vaccination arm receive the RSV vaccine at the start of the model. Each year, individuals in both arms are at risk of developing a symptomatic RSV-ARI episode, which either requires medical care (“medically attended”) or does not (“non-medically attended”). Medical care either consists of “outpatient” (including primary care physician consultations or emergency department visits) or “inpatient” care (hospitalizations). Hospitalized patients can either die (“inpatient mortality”) or be discharged (“no death”). Those in the no-vaccination arm follow the same sequence of events as those in the vaccination arm but with different incidence rates. All individuals are subject to all-cause mortality throughout the time horizon.

### 2.2. Assumptions Related to Vaccination

The vaccination impact was simulated based on a generic RSV vaccine efficacy profile, drawing from published efficacy data from the RSV vaccines evaluated in adults ≥60 years in late-stage development [20,21,22]. The model assumes three profiles of duration of vaccine protection for a single-dose RSV vaccine: one, three, and five years, to test the effect of duration of protection on RSV vaccine outcomes. Based on the reported RSV vaccines’ efficacy in adults ≥60 years old against any symptomatic RSV-ARI, the efficacy for the first year was assumed to be at 70% [20,21,22]. For at least one of the candidate vaccines, sustained vaccine efficacy was observed [21], which informed the three-year duration of protection profile and the efficacy in years 2 and 3. In the absence of observed vaccine efficacy data against symptomatic RSV-ARI for years 4 and 5, some waning of vaccine efficacy was assumed for the five-year duration profile. Vaccine efficacy inputs for the three vaccine profiles are summarized in Table 1.

An assumed vaccination coverage of 59.1% was based on the 2018 seasonal influenza vaccination rates among older adults (≥65 years) in Belgium [23]. Vaccination-associated local and systemic adverse events were obtained specifically from one of the trials [21] (Supplementary Data S1, Table S1-2a to S1-2c).

### 2.3. Model Input Data

The probabilities of events, as well as the associated costs and health input data, were obtained for Belgium through a targeted literature review (Table 2). Demographic data were obtained from Statbel [24]. Costs were converted to 2022 euros using the Health Index from Statistics Belgium [25]. A discount rate of 3% was applied for costs as recommended by Belgian guidelines [26].

#### 2.3.1. Epidemiological Parameters

The incidence rate of symptomatic RSV-ARI and the probability of medical attendance was a pooled estimate derived from Korsten et al. [8] (Supplementary Data S1). This prospective, observational cohort study assessed the community burden of RSV in adults ≥60 years in Europe (Belgium, the United Kingdom, and the Netherlands) from 2017 to 2019. People with symptomatic RSV-ARI included in the study had to present at least one of the pre-defined respiratory symptoms, confirmed by either RT-PCR or serology [8].

The conditional probability of hospitalization for symptomatic RSV-ARI was calculated based on the hospitalization rates for RSV of adults ≥65 years in the Netherlands [27], which is considered comparable to the Belgian context (Supplementary Data S1).

Mortality among hospitalized RSV-positive older adults (≥60 years) was based on inpatient mortality reported from three Belgian hospitals during the 2018–2019 season [28]. Incidence rates of local and systemic adverse events of RSV vaccination were derived from one of the late-stage trials [21].

#### 2.3.2. Cost Parameters

Hospitalization costs were based on the Belgian All Patient Refined Diagnosis Related Groups (APR-DRG) 138. APR-DRG costs represent the amounts reimbursed by compulsory health insurance per hospitalization (i.e., at least one overnight stay), which included daily rates for the hospital stay (including ICU), pharmaceuticals, and other fees (Supplementary Data S1) [29].

The costs of treatment for a medically attended but non-hospitalized and a non-medically attended RSV episode were based on Belgium-specific direct costs from Mao et al. [30] (see details in Supplementary Data S1).

Productivity loss of older adults in Belgium treated in the hospital was calculated using the human capital approach based on the mean length of stay [29], average daily wage [32], and employment rate in the population ≥65 years of age [33]. Workdays lost in ambulatory patients with influenza-like illness (ILI) were applied to medically attended RSV patients who did not require hospitalization [34] (Supplementary Data S1, Table S1-1a and S1-1b). Productivity loss for informal caregivers was calculated using the average daily wage for individuals ≥20 years of age [32], employment rates [33], and workdays lost to care for patients who require hospitalization or ambulatory visits [34] (Supplementary Data S1, Table S1-1c).

Incidence rates of local and systemic adverse events of RSV vaccination were combined with unit cost estimates taken from studies evaluating influenza vaccines [31] to determine average costs of RSV-related adverse events per vaccinated older adult (Supplementary Data S1, Table S1-2a to S1-2c).

### 2.4. Public Health and Economic Outcomes

The public health and economic outcomes were calculated with and without vaccination. RSV-ARI-related public health outcomes included averted number of symptomatic cases, medically attended cases, hospitalizations, and deaths. Complications including pneumonia [35,36,37] and cardiovascular events as reported in previous studies [36,37,38,39] have also been accounted for (Supplementary Data S1). The economic outcomes included savings related to RSV-ARI treatment costs, divided into hospital and outpatient costs. Furthermore, the number needed to vaccinate (NNV) to prevent one RSV-ARI was calculated by dividing the number of vaccinations by each of the public health outcomes.

### 2.5. Sensitivity and Scenario Analyses

Deterministic and probabilistic sensitivity analyses were conducted for the three-year duration of protection vaccine to test the robustness and sensitivity of the model outputs to varying assumptions.

One-way sensitivity analysis was conducted using 95% confidence interval estimates for number of RSV-related hospitalizations prevented and total RSV-associated costs averted. Results were plotted in tornado diagrams.

A probabilistic sensitivity analysis (PSA) was conducted to account for the overall uncertainty of model inputs. For probabilities, a beta distribution was applied. For costs, a gamma distribution was used (Table 2). The minimum and maximum values, standard deviation, and different percentiles for the outputs were obtained from 1000 PSA iterations.

Additionally, a series of scenario and subgroup analyses were conducted. First, following Korsten et al. [8], age groups were stratified (60–74 years and ≥75 years) for all three duration of protection profiles (Supplementary Data S1, Table S1-1d). A long-term scenario was tested, with vaccine efficacy waning by a relative 19% decrease annually compared to the previous year, from the sixth to tenth year [40] (Supplementary Data S1, Figure S1-1). Scenario analyses were also conducted for the three-year duration of protection vaccine to assess the impact of applying a societal perspective which included productivity loss for both patients and caregivers, the effect of different discount rates on costs (0% and 5%), and a conservative vaccination coverage rate of 21.5% informed by pneumococcal vaccination rate [41].

### 2.6. Model Verification and Validation

In line with standard technical validation protocols [42], an internal validation was conducted on the logic of the model structure, mathematical formulas, and data values supplied as model inputs. Additionally, “black-box testing” or extreme value testing can be found in Table S1-3 of Supplementary Data S1. Parallel to this effort, the Excel model was independently replicated in R software, which consistently led to similar results.

## 3. Results

The model estimated a high RSV disease burden in Belgium with 147,232 symptomatic RSV-ARI cases per year, of which 44,988 require medical attention and 3509 require hospitalization, reflecting annual direct medical costs of EUR 35.2 million (Table 3).

The public health outcomes of RSV vaccination are shown in Table 3. Compared with no vaccination, the vaccine with a one-year duration of protection would prevent 60,910 symptomatic RSV-ARI cases and 1452 RSV disease-related hospitalizations. This further translates into the prevention of up to 958 cases of pneumonia, 319 cases of cardiovascular events, 421 cases of sepsis, and 276 cases of acute renal failure (Table 3 and Supplementary Data S2, Table S2-1), derived from the avoided RSV hospitalizations.

Compared with no vaccination, the three-year and five-year duration of protection profiles increased the number of prevented symptomatic RSV-ARI cases up to 154,728 and 224,601, respectively. A similar trend was observed in prevented RSV-associated hospitalizations, complications, and deaths (Table 3 and Supplementary Data S2, Table S2-1).

The one-year duration of protection vaccine is expected to yield EUR 14,547,344 in direct medical cost savings (Table 3). Cost savings increased to EUR 35,982,857 and to EUR 51,033,613 in the three-year and five-year duration of protection profiles, respectively. NNVs to prevent one symptomatic RSV-ARI case ranged from 8 to 28 for the five-, three-, and one-year duration of protection profiles (Table 3). When stratifying the population by age group (Supplementary Data S2, Table S2-2), the NNV to prevent one RSV-ARI case was 10 in adults ≥75 years and 12 in adults 60–74 years for the three-year profile.

**Figure 2:**
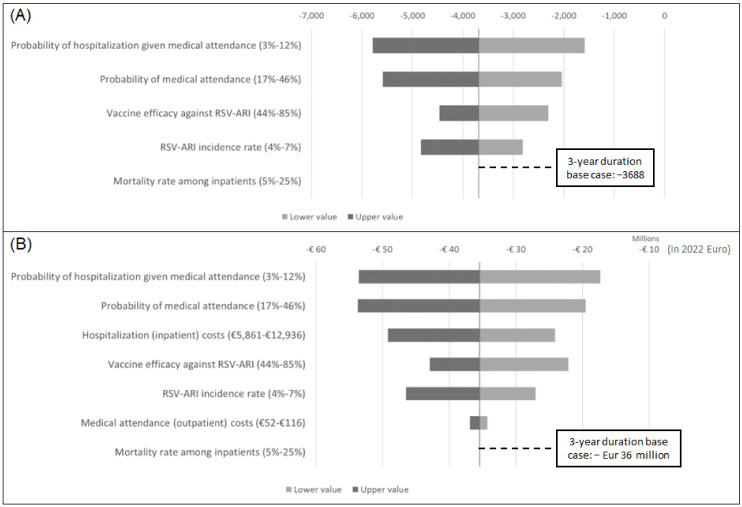
Tornado diagrams for (**A**) incremental number of RSV-related hospitalizations and (**B**) incremental total treatment costs (2022 euros).

The one-way sensitivity analysis showed the impact of several parameters on the number of RSV-related hospitalizations prevented, which was most sensitive to the probabilities of hospitalization and medical attendance, vaccine efficacy, and incidence of symptomatic RSV-ARI (Figure 2A). RSV-associated direct medical costs averted were most sensitive to changes in probabilities of hospitalization and medically attended patients (Figure 2B). Other influential parameters included hospitalization costs, vaccine efficacy, and incidence of symptomatic RSV-ARI.

Table 4 shows the results of the PSA (Monte Carlo simulation of 1000 iterations). The three-year duration of protection vaccine would yield a mean reduction of 153,704 symptomatic RSV-ARI cases, 46,721 medically attended RSV-ARI cases, 3609 hospitalizations, and 495 RSV-attributable deaths and cost savings of EUR 35,091,821 (Table 4).

Scenario analyses of long-term vaccine efficacy showed that there were additional public health benefits and cost savings when vaccine efficacy wanes progressively over five years beyond the assumed vaccine duration of protection. An additional 85,528 symptomatic RSV-ARI cases and EUR 17 million RSV-associated costs would be averted, compared to the five-year protection duration only (Supplementary Data S2, Table S2-3).

When indirect costs were included, expected cost savings increased to EUR 49,696,337 in the scenario analysis of the three-year duration of protection profile (Supplementary Data S2, Table S2-4). Further, applying a discount rate of 0% increased potential cost savings to EUR 37,496,096, while one of 5% decreased potential cost savings to EUR 35,042,428 (Supplementary Data S2, Table S2-5 and S2-6).

Reducing the 59% vaccination coverage to 21.5% for the three-year duration of protection vaccine profile decreased the number of symptomatic RSV-ARI cases and hospitalizations averted to 56,287 and 1342, respectively, and the total cost savings to EUR 13,089,727 (Supplementary Data S2, Table S2-7).

## 4. Discussion

This study showed that RSV vaccination of adults ≥60 years in Belgium, based on the assumed vaccine efficacy, duration of protection, and vaccination coverage, would be expected to result in not only substantial reductions in symptomatic RSV-ARI cases and RSV disease-related hospitalizations, but also savings in direct medical costs. Averting RSV disease-related hospitalizations is further associated with a reduction in complications such as cardiovascular events.

Beyond the positive impact of vaccination on the RSV disease burden, there could be additional benefits in freeing up the health system’s capacity to enable other services [43]. With influenza and RSV circulating during the winter season in addition to SARS-CoV-2, a reduction of 3509 hospitalizations (translating into a decrease of 17,714 bed days) following RSV vaccination would improve hospital capacity in the winter season significantly. In addition, considering that 18% [44] of older adults hospitalized due to RSV require intensive care and 21% [36] require ventilation support, RSV vaccination would significantly improve intensive care capacity.

To assess the impact of different factors on the public health and economic impact of an RSV vaccine, extensive sensitivity and scenario analyses were performed. Duration of vaccine protection was the most important vaccine attribute driving its potential for public health impact. An RSV vaccine that protects against RSV for five years would decrease the number of hospitalizations by 5353 and generate cost savings of EUR 51 million, compared with 3688 avoided hospitalizations and EUR 36 million cost saving with the three-year profile and 1452 prevented hospitalizations and EUR 15 million cost saving with the one-year profile.

Through deterministic sensitivity analysis, other key variables identified were probability of hospitalization and related costs, probability of medical attendance, vaccine efficacy, and symptomatic RSV-ARI incidence. The probabilistic sensitivity analysis suggested that, with a reasonable degree of confidence, the public health impact of an RSV vaccine is significant.

One of the scenario analyses included the societal perspective, in which indirect costs associated with productivity loss of individuals as well as caregivers were included. In this scenario, the economic impact of an RSV vaccine may be increased by almost EUR 14 million for the three-year vaccine profile. The estimated high productivity loss of informal caregivers aligns with the reported high number of informal caregivers in Belgium [45].

To ensure the external validity of this study, the results of this model with similar efficacy were compared with the results of published RSV models using normalized outcomes. Herring et al. [16] reported 5.6 avoided medically attended cases per 100,000 population in the US, while Zeevat et al. [17] estimated 0.07 prevented deaths per 100,000 Dutch population; in comparison, the numbers predicted by this model are 6.4 avoided medically attended cases and 0.07 prevented deaths per 100,000 population, respectively, which are comparable results.

This study further assessed the positive effect of different duration of protection profiles. The NNV to prevent one symptomatic RSV-ARI case is 28 for a duration of one year of protection, which is in line with the reported NNV to prevent one influenza case in older adults [46]. When the duration of protection was extended to three and five years, the NNV was reduced to 11 and 8, respectively, highlighting the improved public health impact that a longer duration of protection confers.

A scenario analysis in this study stratified adults 60–74 years and ≥75 years, estimating the NNV to prevent one RSV-ARI case to be 10 in ≥75-year-olds compared to 12 in adults 60–74 years with a three-year vaccine protection profile. This suggests a similar efficiency of the vaccination program (number of vaccines given to prevent one adverse outcome) regardless of which of the two age groups is targeted. A similar trend between the two age groups was seen in a UK study that demonstrated vaccinating adults ≥65 years and adults ≥75 years would avert a comparable number of RSV-ARI cases per 1000 vaccines [47].

The current study has several limitations. Firstly, while several vaccines have reported efficacy against any symptomatic RSV for one year [20,21,22], only one study [21] has reported vaccine efficacy beyond one year, which was used to populate that specific part of our model. Secondly, the rate of infection was assumed to be constant, meaning that the indirect impact of herd protection was not considered. However, it can be expected that a model accounting for herd protection would result in an even more favorable public health and economic impact of RSV vaccination [48]. Thirdly, this study assumed the same vaccine efficacy in preventing all outcomes from infection, while it is generally observed that vaccine efficacy is greater in preventing more severe outcomes such as severe disease or hospitalization [20,21,22]. Adjusting for this would have resulted in a greater impact. Fourthly, the current analysis did not consider possible RSV-related deaths in the outpatient setting nor the long-term economic impact of an RSV episode. In addition, the probability of hospitalization was calculated based on a Dutch study [27], but the RSV hospitalization pattern is expected to be comparable in Belgium and should not affect study outcomes. Moreover, due to limitations in the literature informing the input parameters stratified by age groups, e.g., probabilities of medical attendance and hospitalization given a symptomatic infection as well as vaccine efficacy and durability of protection for each age group, the age subgroup analysis can be further improved in future research when more detailed data become available. Lastly, this study concerned Belgium, which is believed to be a representative Western European country in terms of economic development, geographic location, and development of healthcare systems [49]. Therefore, the results should be reasonably generalizable to other Western European countries with seasonal RSV circulation.

Future research could consider a projection of dynamic RSV transmission over a longer time horizon and hence the indirect protection of vaccination, which is expected to provide a more holistic picture of the public health impact of RSV vaccination. Moreover, given that about 10% of older adults reside in long-term care facilities in Belgium [45] and the special viral transmission pattern in this setting, exploring the RSV vaccination impact in this population via modeling could be worthwhile.

## 5. Conclusions

The results from this study suggest that RSV vaccination would lead to a substantial decrease in the public health and economic burden of RSV diseases in the adult population ≥60 years in Belgium. The benefits of RSV vaccination also increase when a longer duration of vaccine protection is assumed and when costs of productivity loss are considered. Future research could be directed toward dynamic RSV transmission modeling and settings with special viral transmission patterns.

## Figures and Tables

**Table 1:** RSV vaccine efficacy against any symptomatic ARI.

Vaccine Profile	Vaccine Efficacy against Any Symptomatic ARI (%)				
	Year 1	Year 2	Year 3	Year 4	Year 5
One-year duration	70	-	-	-	-
Three-year duration	70	57	57	-	-
Five-year duration	70	57	57	46	46

-, not applicable; ARI, acute respiratory tract infection.

**Table 2:** Input parameter values.

Description	Value	95% CI	Distribution	References
Symptomatic RSV-ARI incidence rate (≥60 years)	5%	4–7%	Beta	[8]
Probability of medical attendance among symptomatic RSV-ARI cases	31%	17–46%	Beta	[8]
Probability of hospitalization among medically attended cases	8%	3–12%	Beta	[8,27]
Inpatient mortality rate	14%	5–25%	Beta	[28]
Cost per hospitalization	EUR 9057	EUR 5861–12,936	Gamma	[29]
Cost per outpatient treatment	EUR 81	EUR 52–116	Gamma	[30]
Cost associated with non-medically attended symptomatic ARI episode (medication)	EUR 5	EUR 3–7	Gamma	[30]
Cost per vaccination-related local adverse events	EUR 0.02	EUR 0.011–0.024	Gamma	[31]
Cost per vaccination-related systemic adverse events	EUR 0.17	EUR 0.11–0.24	Gamma	[31]
Cost associated with productivity loss for a hospitalized patient	EUR 41	EUR 27–59	Gamma	[29,32,33]
Cost associated with productivity loss for a medically attended, non-hospitalized patient	EUR 21	EUR 13–30	Gamma	[32,33,34]
Caregiver productivity loss associated with a hospitalized patient	EUR 447	EUR 289–693	Gamma	[32,33,34]
Caregiver productivity loss for a medically attended, non-hospitalized patient	EUR 224	EUR 145–319	Gamma	[32,33,34]

CI, confidence interval; RSV-ARI, respiratory syncytial virus-associated acute respiratory tract infection. Costs are expressed in 2022 euros.

**Table 3:** Expected health and economic outcomes for different vaccine profiles in older adults (≥60 years).

	One-Year ^b^			Three-Year ^c^			Five-Year ^d^		
	No Vaccination	Vaccination	Incremental	No Vaccination	Vaccination	Incremental	No Vaccination	Vaccination	Incremental
**Number vaccinated ^a^**	-	1,729,516	1,729,516	-	1,729,516	1,729,516	-	1,729,516	1,729,516
**Symptomatic RSV-ARI cases**	147,232	86,322	−60,910	425,786	271,058	−154,728	682,947	458,345	−224,601
**Medically attended RSV cases**	44,988	26,376	−18,611	130,101	82,823	−42,278	208,678	140,050	−68,628
**RSV hospitalizations**	3509	2057	−1452	10,149	6461	−3688	16,278	10,925	−5353
**RSV-attributable deaths**	477	280	−197	1380	879	−502	2214	1486	−728
**RSV-related treatment costs**	EUR 35,163,993	EUR 20,616,649	EUR −14,547,344	EUR 98,809,396	EUR 62,826,540	EUR −35,982,857	EUR 154,202,095	EUR 103,168,482	EUR −51,033,613
**Inpatient**	EUR 31,315,416	EUR 18,360,228	EUR −12,995,188	EUR 88,014,750	EUR 55,962,919	EUR −32,051,831	EUR 137,355,954	EUR 91,897,618	EUR −45,458,336
**Outpatient**	EUR 3,312,116	EUR 1,941,893	EUR −1,370,222	EUR 9,308,994	EUR 5,918,991	EUR −3,390,004	EUR 14,527,631	EUR 9,719,671	EUR −4,807,960
**Non-medically attended**	EUR 536,461	EUR 314,527	EUR −221,934	EUR 1,485,652	EUR 944,631	EUR −541,021	EUR 2,318,511	EUR 1,551,193	EUR −767,318
**Vaccination-associated adverse event costs**	EUR 0	EUR 320,980	EUR 320,980	EUR 0	EUR 320,980	EUR 320,980	EUR 0	EUR 320,980	EUR 320,980
**Number needed to vaccinate to prevent one**									
**Symptomatic RSV-ARI case**			28			11			8
**Medically attended RSV case**			93			37			25
**RSV hospitalization**			1191			469			323
**RSV-attributable death**			8760			3448			2376

-, not applicable. ^a^ Numbers are rounded; ^b^ one-year duration of protection at 70% vaccine efficacy; ^c^ three-year duration of protection at 70% vaccine efficacy in year 1 and 57% in years 2 and 3; ^d^ five-year duration of protection with vaccine efficacy of 70% in year 1, 57% in years 2 and 3, and 46% in years 4 and 5. RSV-ARI, respiratory syncytial virus-associated acute respiratory tract infection.

**Table 4:** Results of the probabilistic sensitivity analysis for an RSV vaccine with three-year duration of protection *.

	Results			
	Mean	25th Percentile ^a^	50th Percentile (Median) ^b^	75th Percentile ^c^
**Symptomatic RSV-ARI averted**	153,704	138,871	153,221	167,639
**Medically attended RSV-ARI averted**	46,721	36,761	45,390	55,561
**RSV hospitalizations averted**	3609	2475	3409	4434
**RSV-attributable deaths averted**	495	290	446	634
**RSV-related treatment costs averted**	EUR 35,091,821	EUR 23,290,543	EUR 32,369,949	EUR 43,781,160

RSV, respiratory syncytial virus. * Assumes duration of protection of three years; ^a^ 25% of the 1000 iterations fall below the value in the column and 75% above; ^b^ 50% of the iterations fall below the value in the column; ^c^ 75% of the iterations fall below the value in the column, and 25% above.

## Data Availability

No new data were created, and data sources used in analysis were presented in this study. Further data sharing is not applicable to this article.

## Supplementary Materials

The following supporting information can be downloaded at: https://www.mdpi.com/article/10.3390/vaccines11050990/s1: Supplementary Data S1: supplementary information on methods [50,51,52,53]; Supplementary Data S2: supplementary information for additional results.
