# Supplementary material for: Predicted Public Health and Economic Impact of Respiratory Syncytial Virus Vaccination with Variable Duration of Protection for Adults ≥60 Years in Belgium
Source: Vaccines (Basel). 2023 May 16;11(5):990. doi: 10.3390/vaccines11050990 (PMC10223592; doi:10.3390/vaccines11050990)
Supplement: Supplementary file 1 [file vaccines-11-00990-s001.zip › Vaccines-2357696_Supplementary Data_Proofread/RSV Vx CEM_Supplementary Data S1_vF.docx]

Supplementary Data S1

**Predicted public health and economic impact of respiratory syncytial virus vaccination with variable duration of protection for adults ≥60 years in Belgium**

**Authors:** Maarten J. Postma^1,2^, Chih-Yuan Cheng^3^, Nasuh C. Buyukkaramikli^3^, Luis Hernandez Pastor ^3^, Ine Vandersmissen ^4^, Thierry Van Effelterre^3^, Peter Openshaw^5^, Steven Simoens^6^

**Affiliations:**

1. Department of Health Sciences, Unit of Global Health, University of Groningen, University Medical Center Groningen, Groningen, The Netherlands
2. Department of Economics, Econometrics & Finance, University of Groningen, Faculty of Economics& Business, Groningen, The Netherlands
3. Janssen Pharmaceutica NV, Beerse, Belgium
4. Janssen-Cilag NV, Beerse, Belgium
5. National Heart and Lung Institute, Imperial College London, London, UK
6. Department of Pharmaceutical and Pharmacological Sciences, KU Leuven, Leuven, Belgium

**Model input data - background information**

**RSV-ARI incidence rate**

In a prospective study by Korsten et al., participants in an ambulatory setting were contacted weekly during the RSV season to survey for symptoms of acute respiratory tract infection (ARTI). The symptoms include cough, nasal congestion or discharge, wheezing or shortness of breath. Patients who reported any of the symptoms lasting for at least one day were systematically tested for RSV using both RT-PCR and serology. The authors reported an RSV-illness incidence rate of 4.2% in the 2017-2018 season and 7.2% in the 2018-2019 season [1]. This is similar to the incidence reported earlier in the United States [2].

The symptomatic RSV-ARI incidence rate of 5% used in the model was obtained by pooling the observations of the symptomatic cases (88% out of all RSV-illness) from different seasons using an inverse variance method and a fixed effects model. Afterwards, corresponding beta distribution parameters were fitted to match the estimates (mean as well as range of the 95% confidence interval).

**Probability of medical attendance**

In the study by Korsten et al., it was observed that 11 out of 36 older adults with PCR-confirmed RSV-ARTI visited or contacted their general practitioner over the course of two seasons. Based on these data, the probability that an individual with symptomatic RSV-ARI would seek medical attendance was estimated at 31% [1].

**Probability of hospitalization**

No Belgium-specific literature was found that described the probability of hospitalization or hospitalization rates for RSV-ARI. The probability of an individual being hospitalized after experiencing medically attended RSV-ARI was calculated based on the RSV-associated hospitalization rates of older adults (≥65 years) in the Netherlands [3]. To calculate the probability of hospitalization given medical attention, the hospitalization rates were combined with RSV incidence and medical attendance reported by Korsten et al. [1]

**Complications in hospitalized individuals**

Studies have shown that older adults who are hospitalized with RSV disease are subject to increased risk of complications including pneumonia (40-66% of patients) [4-6], cardiovascular events including myocardial infarction (14-22%) [5-8], bacteremia/sepsis (29%) [5], and acute renal failure (19%) [5, 9]. To estimate the number averted of each of these complications, the number of hospitalizations averted per vaccine profile was multiplied by these percentages.

**Hospitalization (inpatient) costs**

The inpatient hospitalization costs consist of the average sums reimbursed by compulsory health insurance per hospitalization (i.e., with minimum one overnight stay), based on Belgian All Patient Refined Diagnosis Related Groups (APR-DRG) 138 [10]. This is a weighted average composed of daily hospital rates (including both ward and ICU stays), pharmaceuticals, and fees. For older adults (≥60 years), the mean costs per day were €681.36 and the mean invoiced length of stay was 12.2 days in 2019 [10].

**Medically attended (outpatient) costs**

The costs for the treatment of a non-hospitalized, medically attended RSV episode were based on Belgium-specific direct costs reported for medically attended RSV patients (n=11) in the European RESCEU observational cohort study [11]. These costs included the costs for health care visits and medication from the Belgian healthcare payer perspective.

**Non-medically attended costs**

The direct medical costs for non-medically attended RSV (i.e., costs for medication) were also based on the RESCEU study (n=25 non-medically attended RSV patients) [11]. These costs included the costs for medication from the Belgian healthcare payer perspective.

**Productivity loss**

Productivity loss per person for older adults treated in the hospital was calculated based on the median length of stay for older RSV patients (≥65 years) (12.2 days, based on the mean invoiced length of stay for RSV patients in 2019 [10], the average daily wage in Belgium for older adults (≥60 years) in 2019 (€163) [12] and the employment rate in Belgium for the population ≥65 years of age (2.9%) in 2020 [13] (Table S1-1a & S1-1b).

For non-hospitalized, medically attended RSV patients, productivity loss was calculated based on workdays lost to ambulatory influenza-like illness in patients of all ages (4 days;(based on data obtained in the same survey conducted among a sample of the general population in Belgium in 2011-2012 [14]), the average daily wage [12], and employment rate [13] in the older Belgian population (≥65 years) (Table S1-1a & S1-1b).

Similar methods were applied to calculate the productivity loss for informal caregivers. We calculated weighted average daily wages for the Belgian population ≥20 years of age (€68) based on the demographic distribution [15], age-specific average monthly wages [12] and employment rates [13] (Table S1-1a & S1-1c). Workdays lost due to care for patients who require hospitalization was assumed to be 6 days, and that for non-hospitalized, medically attended RSV patients was assumed to be 3 days [14] (Table S1-1c).

**Description of scenario analyses**

In the age subgroup analysis, age groups were stratified (60-74 years and ≥75 years), and the relative risk of 0.93 was derived comparing the RSV incidence of age 60-74 years and that of age ≥75 years [1] (Table S1-1d). We applied the relative risk weighted by the size of population in each age group for all other epidemiological parameters to calculate results by age group for all three duration of protection profiles. Regarding to testing long-term vaccine efficacy beyond five years, we analyzed in a scenario where the vaccine efficacy wanes by 19% annually over the next five years beyond the fifth year (Figure S1-1).

**Table S1-1a. Demographics, average monthly wages, and employment rates by age group in Belgium**

| **Age** | **Proportion of population of the age group among all population ≥20 years** | **Average monthly wages** | **Employment rates** |
| --- | --- | --- | --- |
| **20-24** | 7.5% | 2,545 | 38.9% |
| **25-29** | 8.3% | 2,952 | 75.7% |
| **30-34** | 8.3% | 3,365 | 80.6% |
| **35-39** | 8.4% | 3,711 | 82.2% |
| **40-44** | 8.2% | 4,000 | 82.7% |
| **45-49** | 8.6% | 4,099 | 82.6% |
| **50-54** | 8.8% | 4,214 | 78.1% |
| **55-59** | 9.0% | 4,510 | 70.6% |
| **60-64** | 8.3% | 4,904 | 34.3% |
| **65+** | 24.7% | 4,904 | 2.9% |
| **Source** | [15] | [12] | [13] |

**Table S1-1b. Calculation of productivity loss for patients: medically attended and hospitalized and medically attended**

|  | **Value** | **Source** |
| --- | --- | --- |
| Average monthly wage per person in Belgium for 60+ (in Euros) | 4,904 | [12] |
| Average daily wage per person in Belgium for 60+ (in Euros) | 163.5 | Calculated |
| Employment rate in Belgium for 65+ population | 2.9% | [13] |
| CPI for health in January 2019 | 108.5 | [16] |
| CPI for health in January 2020 | 109.7 | [16] |
| CPI for health in January 2022 | 118.2 | [16] |
| Workdays lost due to ILI | 4 | [14] |
| Productivity loss due to ILI per person (in Euros) - 2019 | 19.0 | Calculated |
| Adjusted productivity loss due to ILI per person (in Euros) - 2022 | 20.7 | Calculated |
| LOS for RSV (days) | 12.2 | [10] |
| Productivity loss due to influenza-related hospitalization (in Euros) – 2019 | 57.8 | Calculated |
| Adjusted productivity loss due to influenza-related hospitalization (in Euros) – 2022 | 63.0 | Calculated |

**Table S1-1c. Calculation of productivity loss for informal caregivers: medically attended and hospitalized and medically attended**

|  | **Value** | **Source** |
| --- | --- | --- |
| Weighted average monthly wage per person in Belgium for 20+, adjusted with demographic distribution and age-specific employment rates (in Euros) | 2,053 | Calculated |
| Weighted average daily wage per person in Belgium for 20+ (in Euros) | 68.4 | Calculated |
| CPI for health in January 2019 | 108.5 | [16] |
| CPI for health in January 2022 | 118.2 | [16] |
| Workdays lost due to care for patients with ILI | 3 | [14] |
| Productivity loss due to ILI per person (in Euros) - 2019 | 205.3 | Calculated |
| Adjusted productivity loss due to ILI per person (in Euros) - 2022 | 223.6 | Calculated |
| Workdays lost due to care for ILI patients requiring hospitalization | 6 | [14] |
| Productivity loss due to influenza-related hospitalization (in Euros) – 2019 | 410.6 | Calculated |
| Adjusted productivity loss due to influenza-related hospitalization (in Euros) – 2022 | 447.3 | Calculated |

**Table S1-1d. Parameters used in age subgroup analysis**

|  | **60-74 years** | **≥75 years** |
| --- | --- | --- |
| **RSV-ARI incidence rate** | 5.44% | 5.87% |
| **Risk ratio (60-74 years vs. ≥75 years)** | 0.926 | |
| **Population size (%)** | 1,894,138 (64.73%) | 1,032,285 (35.27%) |
| **Weighted risk ratio** | 59.96% | 40.04% |


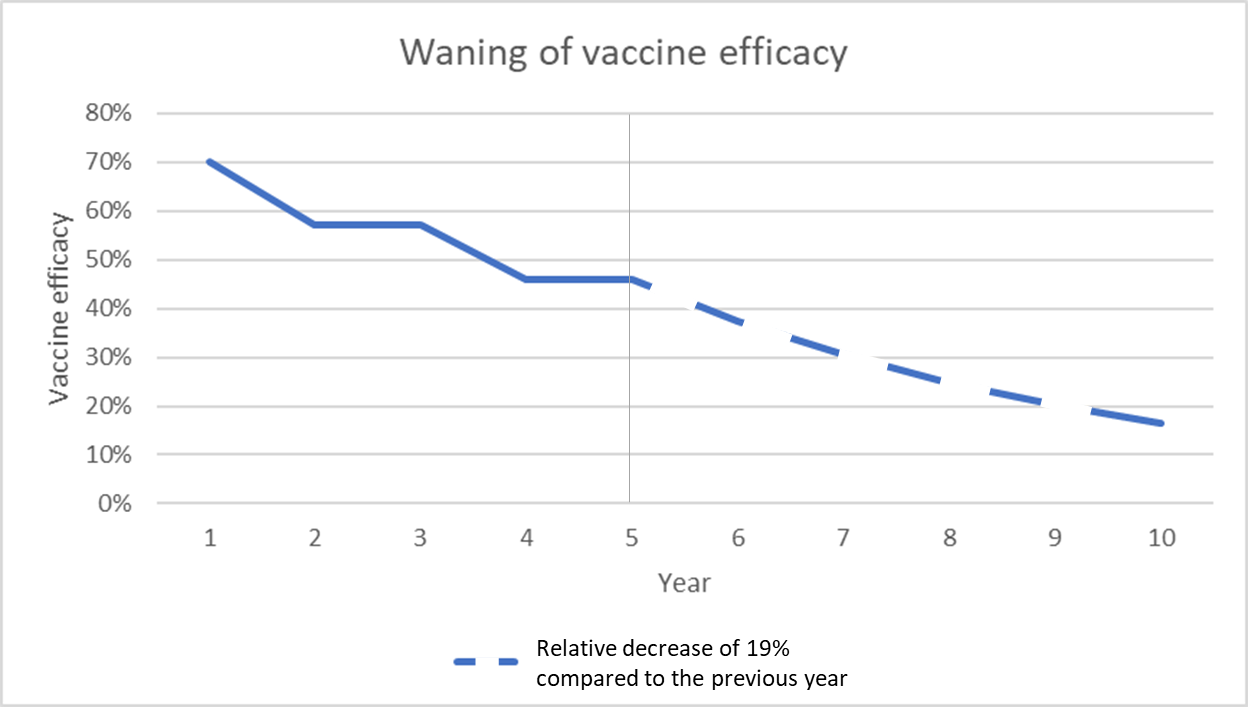


**Figure S1-1. Waning assumptions of vaccine efficacy**

**Calculation of costs for vaccine-related local and systemic adverse events**

The incidence of vaccine-related local and systemic adverse events was observed in the CYPRESS study [17]. The costs per adverse event were informed by the management costs for local and systemic adverse events of flu vaccination [18]. The costs were converted from 2007 USD to 2022 Euro by using the US CPI index (factor 1.558) [19] and the conversion rate from European Central Bank (USD vs. EUR 0.876, 1 year average 24 May 2021 - 24 May 2022) [20].

**Table S1-2a. Grade 3+ Solicited Local Adverse Events (up to 7 days post-vaccination)**

| **Adverse events** | **Incidence** | **Costs per event (2010 USD)** | **Costs per event (2022 Euro)** | **Source** |
| --- | --- | --- | --- | --- |
| Erythema | 1.1% | $ 0.72 | € 0.98 | [17, 18] |
| Pain/Tenderness | 0.6% | $ 0.72 | € 0.98 | [17, 18] |

**Table S1-2b. Grade 3+ Solicited Systemic Adverse Events (up to 7 days post-vaccination)**

| **Adverse events** | **Incidence** | **Costs per event (2010 USD)** | **Costs per event (2022 Euro)** | **Source** |
| --- | --- | --- | --- | --- |
| Fatigue | 1.4% | $ 2.88 | € 3.93 | [17, 18] |
| Headache | 1.4% | $ 2.88 | € 3.93 | [17, 18] |
| Myalgia | 0.6% | $ 2.88 | € 3.93 | [17, 18] |
| Nausea | 0.6% | $ 2.88 | € 3.93 | [17, 18] |
| Pyrexia | 0.3% | $ 2.88 | € 3.93 | [17, 18] |

**Table S1-2c. Final adverse events costs**

|  | **AE costs per vaccinated person per year** |
| --- | --- |
| **Grade 3+ Local AEs** | € 0.02 |
| **Grade 3+ Systemic AEs** | € 0.17 |
| **Total** | € 0.19 |

**Table S1-3. List of black-box tests**

| Checks | Findings |
| --- | --- |
| Set all efficacy data for vaccinated group to 0. LY estimates for both comparators should be the same. | Working as intended. |
| Set all efficacy and safety parameters to be equal between vaccinated and unvaccinated (e.g., set to 0 for the vaccination arm). Check that incremental costs are now mostly (if not exclusively) driven by differences in vaccine acquisition costs (or any other drug-related costs). | Working as intended. |
| Explore the impact of waning with faster drop off in efficacy. Incremental effects would decrease with faster waning. | Working as intended |
| Incremental results with waning would be higher than those without residual waning efficacy. | Working as intended |
| Absolute outcomes should increase with longer duration protection, but relative reduction should slightly decrease (due to impact of natura; death) | Working as intended |
| Set background mortality rates to 1. All patients should die in cycle 1, but still incur vaccine costs. | Working as intended |
| Double the age-specific mortality rates. Number of cases should decrease for both comparators. | Working as intended |
| Set background mortality rate to 0. All patients should survive and still produce economic/health outcomes at the end of the time horizon. | Working as intended |
| Set all AE probabilities to 0. Make sure that no AEs occur, and that AE-related outcomes are also estimated to be 0. | Working as intended |
| Set unit costs for vaccination to 0. Only vaccine administration costs are included. | Working as intended |
| Set vaccine-related unit costs, administration costs, and monitoring costs to 0. Estimated vaccination costs should be 0. | Working as intended |
| Halve and double vaccine unit cost. Estimated undiscounted vaccination costs on the Results sheet should also halve and double in response. | Working as intended |
| Halve, double, and set to 0 vaccine administration costs. Estimated vaccine administration costs on the Results sheet should also halve and double in response. | Working as intended |
| Double the costs per case for each health state (i.e., inpatient, outpatient costs, etc.). RSV costs should increase in the vaccinated arm and unvaccinated arm. The incremental RSV costs (i.e., costs avoided) should increase. | Working as intended |
| Turn indirect costs off or set to 0 while on societal perspective. Costs for productivity loss or caregiver should be 0. | Working as intended |
| Explore longer and shorter time horizons. Health outcomes and total costs should increase/decrease with longer/shorter time horizons. | Working as intended |
| Set the discount rate of health outcomes to 100%. The health outcomes should dramatically decrease. | Working as intended |
| Set the discount rate of costs to 100%. Total costs should dramatically decrease. | Working as intended |
| Set the discount rate of health outcomes to 0%. Total discounted health outcomes should increase and match undiscounted results exactly. | Working as intended |
| Set the discount rate of costs to 0%. Total discounted costs should increase and match undiscounted results exactly. | Working as intended |
| Set lower and upper bound values for all of the inputs equal to the base-case value. The 1-way sensitivity analysis should not have any variation in the tornado diagram. | Working as intended. |

AE = adverse event; RSV = respiratory syncytial virus; VE = vaccine efficacy.

**References**

1. Korsten K, Adriaenssens N, Coenen S, Butler C, Ravanfar B, Rutter H, Allen J, Falsey A, Pircon JY, Gruselle O *et al*: **Burden of respiratory syncytial virus infection in community-dwelling older adults in Europe (RESCEU): an international prospective cohort study**. *Eur Respir J* 2021, **57**(4), doi: 10.1183/13993003.02688-2020.

2. Falsey AR, Hennessey PA, Formica MA, Cox C, Walsh EE: **Respiratory syncytial virus infection in elderly and high-risk adults**. *N Engl J Med* 2005, **352**(17):1749-1759, doi: 10.1056/NEJMoa043951.

3. Jansen AG, Sanders EA, Hoes AW, van Loon AM, Hak E: **Influenza- and respiratory syncytial virus-associated mortality and hospitalisations**. *Eur Respir J* 2007, **30**(6):1158-1166, doi: 10.1183/09031936.00034407.

4. Ieven M, Coenen S, Loens K, Lammens C, Coenjaerts F, Vanderstraeten A, Henriques-Normark B, Crook D, Huygen K, Butler CC *et al*: **Aetiology of lower respiratory tract infection in adults in primary care: a prospective study in 11 European countries**. *Clin Microbiol Infect* 2018, **24**(11):1158-1163, doi: 10.1016/j.cmi.2018.02.004.

5. Tseng HF, Sy LS, Ackerson B, Solano Z, Slezak J, Luo Y, Fischetti CA, Shinde V: **Severe Morbidity and Short- and Mid- to Long-term Mortality in Older Adults Hospitalized with Respiratory Syncytial Virus Infection**. *J Infect Dis* 2020, **222**(8):1298-1310, doi: 10.1093/infdis/jiaa361.

6. Lee N, Lui GC, Wong KT, Li TC, Tse EC, Chan JY, Yu J, Wong SS, Choi KW, Wong RY *et al*: **High morbidity and mortality in adults hospitalized for respiratory syncytial virus infections**. *Clin Infect Dis* 2013, **57**(8):1069-1077, doi: 10.1093/cid/cit471.

7. Volling C, Hassan K, Mazzulli T, Green K, Al-Den A, Hunter P, Mangat R, Ng J, McGeer A: **Respiratory syncytial virus infection-associated hospitalization in adults: a retrospective cohort study**. *BMC Infect Dis* 2014, **14**:665, doi: 10.1186/s12879-014-0665-2.

8. Kwong JC, Schwartz KL, Campitelli MA, Chung H, Crowcroft NS, Karnauchow T, Katz K, Ko DT, McGeer AJ, McNally D *et al*: **Acute Myocardial Infarction after Laboratory-Confirmed Influenza Infection**. *N Engl J Med* 2018, **378**(4):345-353, doi: 10.1056/NEJMoa1702090.

9. Ackerson B, Tseng HF, Sy LS, Solano Z, Slezak J, Luo Y, Fischetti CA, Shinde V: **Severe Morbidity and Mortality Associated With Respiratory Syncytial Virus Versus Influenza Infection in Hospitalized Older Adults**. *Clin Infect Dis* 2019, **69**(2):197-203, doi: 10.1093/cid/ciy991.

10. **Gegevens uit de databank Medische Diagnose/Zorg & Kost, de dato 01 02 2022; Technische Cel voor de verwerking van de gegevens met betrekking tot ziekenhuizen, RIZIV/INAMI, België** [<https://tct.fgov.be/webetct/etct-web/html/nl/index.jsp>] (Accessed 16 August 2022)

11. Mao Z, Li X, Korsten K, Bont L, Butler C, Wildenbeest J, Coenen S, Hens N, Bilcke J, Beutels P *et al*: **Economic Burden and Health-Related Quality of Life of Respiratory Syncytial Virus and Influenza Infection in European Community-Dwelling Older Adults**. *J Infect Dis* 2022, **226**(Supplement_1):S87-S94, doi: 10.1093/infdis/jiac069.

12. **Average gross monthly wages** [<https://data.gov.be/fr/dataset/5632a04bda7d61aa3d52d666fce8d0a963f28130>] (Accessed 16 August 2022)

13. OECD: **Labour Market Statistics: Labour force statistics by sex and age: indicators (Edition 2021)**. In*.*; 2022.

14. Bilcke J, Coenen S, Beutels P: **Influenza-like-illness and clinically diagnosed flu: disease burden, costs and quality of life for patients seeking ambulatory care or no professional care at all**. *PLoS One* 2014, **9**(7):e102634, doi: 10.1371/journal.pone.0102634.

15. **Structure of the Population** [<https://statbel.fgov.be/en/themes/population/structure-population>] (Accessed 16 August 2022)

16. **Consumer price index and health index** [<https://statbel.fgov.be/en/open-data/consumer-price-index-and-health-index>] (Accessed 16 August 2022)

17. Bart S, Williams K, Gymnopoulou E, Falsey AR, Ervin J, Bastian AR, Menten J, De Paepe E, de Boer H, Vandenberghe S *et al*: **Safety and Tolerability of an Ad26.RSV.preF-based Vaccine in a Randomized, Double-blind, Placebo-controlled, Phase 2b Study in Adults Aged ≥65 Years**. In: *8th ESWI Influenza Conference.* Salzburg, Austria; 2021.

18. Lee BY, Ercius AK, Smith KJ: **A predictive model of the economic effects of an influenza vaccine adjuvant for the older adult (age 65 and over) population**. *Vaccine* 2009, **27**(16):2251-2257, doi: 10.1016/j.vaccine.2009.02.024.

19. **Consumer Price Index for All Urban Consumers [CPI-U] Medical Care** [<https://www.bls.gov/cpi/data.htm>] (Accessed 16 August 2022)

20. **US dollar (USD)** [<https://www.ecb.europa.eu/stats/policy_and_exchange_rates/euro_reference_exchange_rates/html/eurofxref-graph-usd.en.html>] (Accessed 24 May 2022)
