# Supplementary material for: Predicted Public Health and Economic Impact of Respiratory Syncytial Virus Vaccination with Variable Duration of Protection for Adults ≥60 Years in Belgium
Source: Vaccines (Basel). 2023 May 16;11(5):990. doi: 10.3390/vaccines11050990 (PMC10223592; doi:10.3390/vaccines11050990)
Supplement: Supplementary file 1 [file vaccines-11-00990-s001.zip › Vaccines-2357696_Supplementary Data_Proofread/RSV Vx CEM_Supplementary Data S2_vF.docx]

Supplementary Data S2

**Predicted public health and economic impact of respiratory syncytial virus vaccination with variable duration of protection for adults ≥60 years in Belgium**

**Authors:** Maarten J. Postma^1,2^, Chih-Yuan Cheng^3^, Nasuh C. Buyukkaramikli^3^, Luis Hernandez Pastor ^3^, Ine Vandersmissen ^4^, Thierry Van Effelterre^3^, Peter Openshaw^5^, Steven Simoens^6^

**Affiliations:**

1. Department of Health Sciences, Unit of Global Health, University of Groningen, University Medical Center Groningen, Groningen, The Netherlands
2. Department of Economics, Econometrics & Finance, University of Groningen, Faculty of Economics& Business, Groningen, The Netherlands
3. Janssen Pharmaceutica NV, Beerse, Belgium
4. Janssen-Cilag NV, Beerse, Belgium
5. National Heart and Lung Institute, Imperial College London, London, UK
6. Department of Pharmaceutical and Pharmacological Sciences, KU Leuven, Leuven, Belgium

**Table S2-1. Expected number of RSV-associated complications averted compared with no vaccination**

|  | **One-year duration of protection** | | **Three-year duration of protection** | | **Five-year duration of protection** | |
| --- | --- | --- | --- | --- | --- | --- |
|  | **Low estimate** | **High estimate** | **Low estimate** | **High estimate** | **Low estimate** | **High estimate** |
| **Pneumonia** | 581 | 958 | 1,475 | 2,434 | 2,141 | 3,533 |
| **Cardiovascular event including myocardial infarction** | 203 | 319 | 516 | 811 | 749 | 1,178 |
| **Bacteremia/Sepsis** | 421 | | 1,070 | | 1,552 | |
| **Acute renal failure** | 276 | | 701 | | 1,017 | |

**Table S2-2. Expected health and economic outcomes for different vaccine profiles by age group, compared with no vaccination**

|  | **Total numbers** | | | | | | **NNV to avert one case** | | | | | | |
| --- | --- | --- | --- | --- | --- | --- | --- | --- | --- | --- | --- | --- | --- |
|  | **One-year duration of protection** | | **Three-year duration of protection** | | **Five-year duration of protection** | | **One-year duration of protection** | | **Three-year duration of protection** | | **Five-year duration of protection** | | |
|  | **60-74 years** | **≥75 years** | **60-74 years** | **≥75 years** | **60-74 years** | **≥75 years** | **60-74 years** | **≥75 years** | **60-74 years** | **≥75 years** | **60-74 years** | | **≥75 years** |
| **Symptomatic RSV-ARI cases averted** | 36,520 | 24,390 | 92,771 | 61,957 | 134,665 | 89,936 | 31 | 25 | 12 | 10 | 8 | 7 | |
| **Medically attended RSV-ARI cases averted** | 11,159 | 7,452 | 25,349 | 16,929 | 41,148 | 27,480 | 100 | 82 | 44 | 36 | 27 | 22 | |
| **RSV hospitalizations averted** | 871 | 581 | 2,211 | 1,477 | 3,210 | 2,143 | 1,286 | 1,049 | 506 | 413 | 349 | 285 | |
| **RSV-attributable deaths averted** | 118 | 79 | 301 | 201 | 436 | 292 | 9,477 | 7,734 | 3,719 | 3,035 | 2,565 | 2,093 | |
| **RSV-related treatment costs averted** | € 8,722,200 | € 5,825,144 | € 21,574,363 | € 14,408,494 | € 30,598,396 | € 20,435,217 | - | - | - | - | - | - | |
| **Inpatient costs** | € 7,791,569 | € 5,203,619 | € 19,217,425 | € 12,834,406 | € 27,255,608 | € 18,202,728 | - | - | - | - | - | - | |
| **Outpatient costs** | € 821,549 | € 548,673 | € 2,032,556 | € 1,357,448 | € 2,882,725 | € 1,925,235 | - | - | - | - | - | - | |
| **Non-medically attended costs** | € 133,066 | € 88,868 | € 324,382 | € 216,639 | € 460,063 | € 307,255 | - | - | - | - | - | - | |
| **Vaccination-associated adverse event management costs** | € 207,755 | € 113,225 | € 207,755 | € 113,225 | € 207,755 | € 113,225 | - | - | - | - | - | - | |

ARI, acute respiratory infection; NNV, number needed to vaccinate; RSV, respiratory syncytial virus.

**Table S2-3. Expected health and economic outcomes by vaccination status in the population ≥60 years in Belgium, considering the vaccine efficacy rate beyond the fifth year wanes 19% annually over the next five years**

|  | **Five-year duration of protection profile** | | | | **Five-year duration of protection profile:**  **Scenario – vaccine efficacy wanes 19% annually over the next 5 years** | | | |
| --- | --- | --- | --- | --- | --- | --- | --- | --- |
|  | **No vaccination** | **Vaccination** |  |  | **No vaccination** | **Vaccination** |  |  |
|  | **Total** | **Total** | **Incremental** | **NNV** | **Total** | **Total** | **Incremental** | **NNV** |
| **Symptomatic RSV-ARI cases averted** | 682,947 | 458,345 | -224,601 | 8 | 1,231,974 | 921,844 | -310,129 | 6 |
| **Medically attended RSV-ARI cases averted** | 208,678 | 140,050 | -68,628 | 25 | 376,436 | 281,675 | -94,762 | 18 |
| **RSV hospitalizations averted** | 16,278 | 10,925 | -5,353 | 323 | 29,364 | 21,972 | -7,392 | 234 |
| **RSV-attributable deaths averted** | 2,214 | 1,486 | -728 | 2,376 | 3,993 | 2,988 | -1,005 | 1,720 |
| **RSV-related treatment costs averted** | € 154,202,095 | € 103,168,482 | € -51,033,613 | - | € 261,196,218 | € 193,298,989 | € -67,897,229 | - |
| **Inpatient costs** | € 137,355,954 | € 91,897,618 | € -45,458,336 | - | € 232,661,272 | € 172,181,622 | € -60,479,650 | - |
| **Outpatient costs** | € 14,527,631 | € 9,719,671 | € -4,807,960 | - | € 24,607,721 | € 18,211,013 | € -6,396,709 | - |
| **Non-medically attended costs** | € 2,318,511 | € 1,551,193 | € -767,318 | - | € 3,927,225 | € 2,906,354 | € -1,020,871 | - |
| **Vaccination-associated adverse event management costs** | €0 | € 320,980 | € 320,980 | - | €0 | €320,980 | €320,980 | - |

ARI, acute respiratory infection; NNV, number needed to vaccinate; RSV, respiratory syncytial virus.

**Table S2-4. Expected health and economic outcomes by vaccination status in the population ≥60 years in Belgium taking a societal perspective, three-year duration of protection vaccine profile**

|  | **Three-year duration of protection vaccine profile** | | | | **Three-year duration of protection vaccine profile:**  **Scenario – societal perspective** | | | |
| --- | --- | --- | --- | --- | --- | --- | --- | --- |
|  | **No vaccination** | **Vaccination** |  |  | **No vaccination** | **Vaccination** |  |  |
|  | **Total** | **Total** | **Incremental** | **NNV** | **Total** | **Total** | **Incremental** | **NNV** |
| **Symptomatic RSV-ARI cases averted** | 425,786 | 271,058 | -154,728 | 11 | 425,786 | 271,058 | -154,728 | 11 |
| **Medically attended RSV-ARI cases averted** | 130,101 | 82,823 | -42,278 | 37 | 130,101 | 82,823 | -47,278 | 37 |
| **RSV hospitalizations averted** | 10,149 | 6,461 | -3,688 | 469 | 10,149 | 6,461 | -3,688 | 469 |
| **RSV-attributable deaths averted** | 1,380 | 879 | -502 | 3,448 | 1,380 | 879 | -502 | 3,448 |
| **RSV-related treatment costs averted** | € 98,809,396 | € 62,826,540 | € -35,982,857 | - | € 98,809,397 | € 62,826,540 | € -35,982,857 | - |
| **Inpatient costs** | € 88,014,750 | € 55,962,919 | € -32,051,831 | - | € 88,014,750 | € 55,962,919 | € -32,051,831 | - |
| **Outpatient costs** | € 9,308,994 | € 5,918,991 | € -3,390,004 | - | € 9,308,994 | € 5,918,991 | € -3,390,004 | - |
| **Non-medically attended costs** | € 1,485,652 | € 944,631 | € -541,021 | - | € 1,485,652 | € 944,631 | € -541,021 | - |
| **Vaccination-associated adverse event management costs** | € 0 | € 320,980 | € 320,980 | - | €0 | €320,980 | €320,980 | - |
| **Indirect costs** | - | - | - | - | € 38,662,493 | € 24,949,012 | € -13,713,480 | - |

ARI, acute respiratory infection; NNV, number needed to vaccinate; RSV, respiratory syncytial virus.

**Table S2-5. Expected health and economic outcomes by vaccination status in the population ≥60 years in Belgium with discount rate for costs at 0%, three-year duration of protection vaccine profile**

|  | **Three-year duration of protection vaccine profile** | | | | **Three-year duration of protection vaccine profile:**  **Scenario – discount rate at 0%** | | | |
| --- | --- | --- | --- | --- | --- | --- | --- | --- |
|  | **No vaccination** | **Vaccination** |  |  | **No vaccination** | **Vaccination** |  |  |
|  | **Total** | **Total** | **Incremental** | **NNV** | **Total** | **Total** | **Incremental** | **NNV** |
| **Symptomatic RSV-ARI cases averted** | 425,786 | 271,058 | -154,728 | 11 | 425,786 | 271,058 | -154,728 | 11 |
| **Medically attended RSV-ARI cases averted** | 130,101 | 82,823 | -42,278 | 37 | 130,101 | 82,823 | -47,278 | 37 |
| **RSV hospitalizations averted** | 10,149 | 6,461 | -3,688 | 469 | 10,149 | 6,461 | -3,688 | 469 |
| **RSV-attributable deaths averted** | 1,380 | 879 | -502 | 3,448 | 1,380 | 879 | -502 | 3,448 |
| **RSV-related treatment costs averted** | € 98,809,396 | € 62,826,540 | € -35,982,857 | - | € 103,182,866 | € 65,686,770 | € -37,496,096 | - |
| **Inpatient costs** | € 88,014,750 | € 55,962,919 | € -32,051,831 | - | € 91,910,431 | € 58,510,677 | € -33,399,754 | - |
| **Outpatient costs** | € 9,308,994 | € 5,918,991 | € -3,390,004 | - | € 9,721,026 | € 6,188,458 | € -3,532,568 | - |
| **Non-medically attended costs** | € 1,485,652 | € 944,631 | € -541,021 | - | € 1,551,410 | € 987,636 | € -563,774 | - |
| **Vaccination-associated adverse event management costs** | € 0 | € 320,980 | € 320,980 | - | €0 | €320,980 | €320,980 | - |

ARI, acute respiratory infection; NNV, number needed to vaccinate; RSV, respiratory syncytial virus.

**Table S2-6. Expected health and economic outcomes by vaccination status in the population ≥60 years in Belgium with discount rate for costs at 5%, three-year duration of protection vaccine profile**

|  | **Three-year duration of protection vaccine profile** | | | | **Three-year duration of protection vaccine profile:**  **Scenario – discount rate at 5%** | | | |
| --- | --- | --- | --- | --- | --- | --- | --- | --- |
|  | **No vaccination** | **Vaccination** |  |  | **No vaccination** | **Vaccination** |  |  |
|  | **Total** | **Total** | **Incremental** | **NNV** | **Total** | **Total** | **Incremental** | **NNV** |
| **Symptomatic RSV-ARI cases averted** | 425,786 | 271,058 | -154,728 | 11 | 425,786 | 271,058 | -154,728 | 11 |
| **Medically attended RSV-ARI cases averted** | 130,101 | 82,823 | -42,278 | 37 | 130,101 | 82,823 | -47,278 | 37 |
| **RSV hospitalizations averted** | 10,149 | 6,461 | -3,688 | 469 | 10,149 | 6,461 | -3,688 | 469 |
| **RSV-attributable deaths averted** | 1,380 | 879 | -502 | 3,448 | 1,380 | 879 | -502 | 3,448 |
| **RSV-related treatment costs averted** | € 98,809,396 | € 62,826,540 | € -35,982,857 | - | € 96,093,973 | € 61,051,544 | € -35,042,428 | - |
| **Inpatient costs** | € 88,014,750 | € 55,962,919 | € -32,051,831 | - | € 85,595,979 | € 54,381,836 | € -31,214,142 | - |
| **Outpatient costs** | € 9,308,994 | € 5,918,991 | € -3,390,004 | - | € 9,053,170 | € 5,751,766 | € -3,301,404 | - |
| **Non-medically attended costs** | € 1,485,652 | € 944,631 | € -541,021 | - | € 1,444,824 | € 917,943 | € -526,882 | - |
| **Vaccination-associated adverse event management costs** | € 0 | € 320,980 | € 320,980 | - | €0 | €320,980 | €320,980 | - |

ARI, acute respiratory infection; NNV, number needed to vaccinate; RSV, respiratory syncytial virus.

**Table S2-7. Expected health and economic outcomes by vaccination status in the population ≥60 years in Belgium with vaccine coverage of 21.5%, three-year duration of protection profile**

|  | **Three-year duration of protection vaccine profile: base case with vaccination coverage 59.1%** | | | | **Three-year duration of protection profile:**  **Scenario – vaccination coverage at 21.5%** | | | |
| --- | --- | --- | --- | --- | --- | --- | --- | --- |
|  | **No vaccination** | **Vaccination** |  |  | **No vaccination** | **Vaccination** |  |  |
|  | **Total** | **Total** | **Incremental** | **NNV** | **Total** | **Total** | **Incremental** | **NNV** |
| **Symptomatic RSV-ARI cases averted** | 425,786 | 271,058 | -154,728 | 11 | 425,786 | 369,500 | -56,287 | 11 |
| **Medically attended RSV-ARI cases averted** | 130,101 | 82,823 | -42,278 | 37 | 130,101 | 112,903 | -17,199 | 37 |
| **RSV hospitalizations averted** | 10,149 | 6,461 | -3,688 | 469 | 10,149 | 8,807 | -1,342 | 469 |
| **RSV-attributable deaths averted** | 1,380 | 879 | -502 | 3,448 | 1,380 | 1,198 | -182 | 3,448 |
| **RSV-related treatment costs averted** | € 98,809,396 | € 62,826,540 | € -35,982,857 | - | € 98,809,397 | € 87,719,670 | € -13,089,727 | - |
| **Inpatient costs** | € 88,014,750 | € 55,962,919 | € -32,051,831 | - | € 88,014,750 | € 76,355,039 | € -11,659,711 | - |
| **Outpatient costs** | € 9,308,994 | € 5,918,991 | € -3,390,004 | - | € 9,308,994 | € 8,075,790 | € -1,233,205 | - |
| **Non-medically attended costs** | € 1,485,652 | € 944,631 | € -541,021 | - | € 1,485,652 | € 1,288,841 | € -196,811 | - |
| **Vaccination-associated adverse event management costs** | € 0 | € 320,980 | € 320,980 | - | € 0 | € 116,769 | € 116,769 | - |

ARI, acute respiratory infection; NNV, number needed to vaccinate; RSV, respiratory syncytial virus.
